# Delayed post-operative contralateral epidural hematoma in a patient with right-sided acute subdural hematoma: a case report

**DOI:** 10.4076/1757-1626-2-6282

**Published:** 2009-08-03

**Authors:** Hooshang Saberi, Ali Tayebi Meybodi, Keyvan Tayebi Meybodi, Zohreh Habibi, Sayed Mohammad Haji Mirsadeghi

**Affiliations:** Department of Neurosurgery, Imam Khomeini Hospital, Tehran University of Medical SciencesTehran 14197Iran

## Abstract

Head injury is one of the leading causes of death and disability in traumatic accidents. Post-operative contralateral epidural hematomas after surgery for acute subdural hematoma seem to be rare. In this case, expansion and spontaneous resolution of a fractural epidural hematoma contralateral to the side of acute subdural hematoma is presented. The importance of immediate post-operative computed tomography is also highlighted to detect delayed traumatic mass lesions.

## Introduction

Despite recent advances in diagnostic and therapeutic modalities, acute SDHs remain among the most lethal traumatic lesion of the brain, with a mortality rate of approximately 50-74% [1]. Outcome has been found to be related to clinical features such as patient’s age, trauma-treatment span, pupil status, presence of lucid interval, associated extracranial injuries, and intracranial hypertension [2,3]. Another important factor influencing the outcome of such patients is the presence and/or evolution of contralateral hematomas. In case of evolution of new hematomas contralateral to the side of acute SDH, the immediate post-operative diagnosis and timely therapeutic intervention are of utmost importance.

## Case presentation

**Figure 1.**
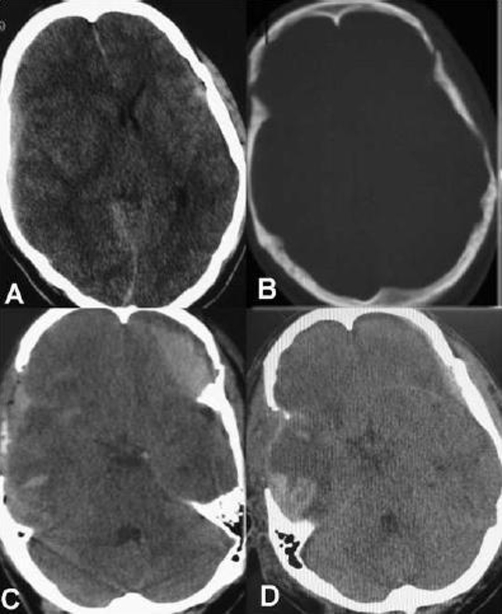
Preoperative axial brain CT, showing right acute subdural hematoma with midline shift and left small EDH **(A)**; axial bone window showing linear skull fracture adjacent to the EDH site **(B)**; post-operative brain CT scan showing expansion of the EDH after decompressive surgery for evacuation of acute subdural hematoma **(C)**; delayed CT scan showing spontaneous resolution of the EDH after 5 days **(D)**.

We report a 19-year-old Caucasian male of Iranian Nationality with acute right-sided traumatic SDH (due to car accident) with presenting Glasgow Coma Scale score of 6/15 and CT evidence of severe brain swelling and associated midline shift and contralateral small fractural EDH in the temporoparietal region (Figure 1). The patient was anisocoric (right sided mydriasis) at presentation. He underwent an emergent decompressive craniectomy and evacuation of the SDH. During surgery, the brain showed considerable swelling after hematoma evacuation and coagulation of the ruptured bridging vein causative of hematoma. Therefore, an augmentation duraplasty using free pericranial flap was performed. Regarding severe brain swelling during operation, a possible intracranial pathology amenable to surgery was suspected and immediate post-operative CT scan revealed expansion of the contralateral EDH. Due to mass effect of EDH, a second surgery was offered. The patient’s family declined second surgery for EDH evacuation. The patient was transferred to intensive care undergoing ICP monitoring. Despite all medical measures such as mannitol, high dose barbiturate therapy, and mild hypothermia, the patient's ICP remained high (about 25 mmHg). CPP was around 65 mmHg. Serial CT scans were performed the next days, showing right PCA territory infarct as well as expansion and spontaneous resolution of the EDH. The ICP remained relatively high (about 20 mmHg) in the following days. The patient was discharged 3 months after admission in persistent vegetative state.

## Discussion

Several neurosurgical procedures have been reported to be associated with the incidence of contralateral EDHs. Some of these procedures include evacuation of acute or chronic SDHs and ventriculo-peritoneal shunt insertion. Different authors have reported the incidence of contralateral EDH after evacuation of ipsilateral acute SDH differently (from very rare to 5.7%) [1,4-6].

Intra-operative brain swelling, post-operative neurologic deterioration, papillary dilation contralateral to the SDH, grand mal seizures, and intractably elevated ICP have been proposed as some hints to the detection of this pathology [5,7]. These signs and symptoms may have more common causes than evolution of contralateral EDH such as: reaccumulation of hematoma and brain edema [6]. On presentation of these clues, immediate CT is recommended, since it may lead to emergent evacuation surgery [1].

Several causes have been proposed for delayed formation/expansion of contralateral EDH including loss of tamponade effect, vasomotor mechanisms, and coagulopathy [8], with the main cause appearing to be the upsetting of the equilibrium of the damaged vessels and the reactive ICP [9]. Possible sources of bleeding include a ruptured meningeal arterial branch (tamponaded because of edema, clot), venous lacerations causing low tension bleeding, or a skull fracture [4]. Some other precipitating factors have been said to be: surgical decompression, CSF fistula, aggressive anti-edema treatment, and systemic hypotension all of which can lead to intracranial hypotension [4].

Paying attention to persistent high or elevating ICP after surgery for evacuation of an acute SDH is critical. Although, most instances of increased ICP after SDH evacuation seem to be due to brain edema, this phenomenon should raise the suspicion of evolution of a contralateral hematoma (as in our case). The presence of skull fractures, though not evident in the initial CT (due to partial volume effects and pixel size) is said to be a prominent risk factor for the malady [5].

Another point to be mentioned is the low incidence of this complication in the elderly, which is elucidated to be due to tight adherence of the dura to skull in this patient population [5]. The diagnostic approach to possible contralateral EDHs is controversial. Some authorities suggest immediate CT in patients who undergo surgery for evacuation of acute SDH that present intraoperative brain swelling [1] or skull fractures [5]. Some authors have proposed to perform burr holes over the fracture during the first surgery [10], but this has been challenged because CT can readily delineate the size and location of the contralateral EDH and dictate the therapeutic policy, and also can detect other intracranial lesions [5].

Prompt detection of acute contralateral EDH in these patients is of critical importance since its treatment will lead to a more efficient ICP management which is directly related to the patient outcome [1]. Less severity of head injury, higher CPP (>70 mmHg), lower ICP, and neurological deterioration due to EDH rather than parenchymal brain injury is related to better long-term outcome in these patients [1,5].

The treatment of contralateral EDH is done according to the CT findings. In the present case, there have been several cues to the expansion of the contralateral EDH: intraoperative brain swelling, contralateral fracture and persistent raised ICP after the first surgery. Second surgery was rejected by the patient’s family, and this revealed the natural course of the malady. It seems that rapid decompression and loss of tamponade effect and upsetting of the ICP equilibrium led to the expansion of the contralateral fractural EDH. Yet, progressive brain swelling and evolution of right PCA infarct causing increased ICP caused expulsion of the hematoma through the fracture line underneath the scalp.

## Conclusion

Expansion/evolution of contralateral hematomas should always be considered after ipsilateral decompression of acute SDH especially in cases of intra-operative brain swelling and skull fractures. A high degree of clinical suspicion is mandatory to confer best outcome to these patients.

## Abbreviations

CPP: Cerebral perfusion pressure
CSF: Cerebrospinal fluid
CT: Computed tomography
EDH: Epidural hematoma
ICP: Intracranial pressure
PCA: Posterior cerebral artery
SDH: Subdural hematoma
